# A longitudinal study of capability-based quality of life and mental health in the first 5-months of lockdown restrictions in the UK

**DOI:** 10.1186/s12889-023-15285-8

**Published:** 2023-03-08

**Authors:** Ross G. White, Paul Christiansen, Catharina van der Boor

**Affiliations:** 1 School of Psychology, Queen’s University Belfast, David Keir Building, 8-30 Malone Road, Belfast, BT9 5BN UK; 2 Institute of Population Health, University of Liverpool, Brownlow Hill, Liverpool, L69 3GB UK; 3 Department for Health Services Research and Policy, London School of Hygiene and Tropical Medicine, 15-17 Tavistock Place, London, WC1H 9SH UK

**Keywords:** COVID19, Mental health, Lockdown, Depression, Anxiety, Capability approach, Quality of life

## Abstract

**Background:**

COVID19, and associated lockdown restrictions, have impacted on people’s daily lives. Understanding the mental health and wellbeing implications of these impacts has been identified as a public health research priority.

**Aims:**

Building on an earlier cross-sectional study, the current study sought to investigate whether capability-based quality of life changed during the first 5-months of lock-down restrictions in the UK, and whether capability-based quality of life was predictive of future levels of depression and anxiety.

**Methods:**

An initial convenience sample of 594 participants were followed up at three different timepoints spanning a 20-week time-period between March 2020 and August 2020. Participants provided demographic information and completed the Oxford Capabilities Questionnaire – Mental Health (OxCAP-MH), and the Hospital Anxiety and Depression Scale (HADS).

**Results:**

The mean scores indicated that levels of both depression and anxiety decreased across the three timepoints, whereas capability-based QoL (as assessed by the OxCAP-MH) decreased over time. Capability-based QoL predicted additional levels of variance in both depression and anxiety levels when time and sociodemographic factors were controlled for. Cross-lagged panel model analyses indicated that capability-based QoL over a month into lockdown restrictions predicted levels of depression and anxiety 5 months into the restrictions.

**Conclusions:**

The study findings suggest that the capability-limiting impact of public health emergencies and related lockdown restrictions are important for understanding peoples’ levels of depression and anxiety. The implications that the findings have for the provision of support in the context of public health emergencies and associated restrictions are discussed.

## Introduction

Since the COVID19 virus was first declared as a public health emergency of international concern in January 2020 [1], the pandemic has subsequently claimed the lives of more than 6.86 million deaths to date worldwide [2].To control the spread of the pandemic, local and national government agencies across the world have implemented a range of different lock-down restrictions and quarantine strategies to slow the spread of the virus and mitigate its effects on healthcare systems and society. These restrictions have had significant impacts on economic activity, education, religious gatherings, sports and culture, and other aspects of people’s social life. A rapid review of 24 studies that investigated the psychological impacts of periods of quarantine (relating to viral outbreaks including SARS, MERS, and Ebola Virus Disease) that predated the COVID19 pandemic [3] found that the majority of studies reported adverse psychological impacts such as post-traumatic stress symptoms, confusion, and anger. Specific stressors associated with quarantine included irregular or lower supplies of food items and medication, restrictions to daily routines, infection fears, and financial loss, amongst others [3]. Several important methodological issues with the research conducted to date merit attention – only one of the studies was longitudinal in nature, the sample sizes of the studies tended to be small, few studies employed control groups with which to compare those who had been quarantined, and (if reported) the periods of quarantine in the studies tended to be considerably shorter than those seen with the COVID19 pandemic [3]. The COVID19 pandemic restrictions differed from previous instances of quarantine, with journeys outside the home of some description still being permissible, advanced warning of the impending lock-down being provided that may have front-ended the psychological impacts, and an array of online-based activities and virtual communication tools being available to potentially buffer against the impact of restrictions for a proportion of the population [4].

Understanding the mental health impact of the COVID19 pandemic and the accompanying restrictions has been identified as a public health research priority [5]. A systematic review and meta-analysis of studies investigating mental health impacts of the COVID19 pandemic included 104 eligible papers – including 43 studies that compared pre-pandemic and pandemic-specific data which were subjected to meta-analysis [6]. The majority of studies had recruited samples from the general population (*n* = 50), with 30 studies focusing on healthcare workers, and 7 studies recruiting patients only (including those experiencing COVID19 and/or pre-existing physical health conditions). A further 17 studies recruited mixed samples. The meta-analysis noted that, relative to pre-pandemic data, levels of anxiety and depression were elevated in the general population in the early phase of the pandemic [6]. However, neither healthcare staff, nor service-users demonstrated elevated levels relative to the pre-pandemic data [6]. Having a pre-existing mental disorder, being female, and having elevated levels of worry about becoming infected were consistently associated with increased risk [6]. On the other hand, being of older age, being economically secure, and having higher levels of education were noted as being protective [6]. Research has also highlighted that the global disease burden associated with symptoms of anxiety and depression (collectively referred to as ‘common mental health difficulties’) have increased considerably during the COVID19 pandemic and its aftermath [7].

Calls have been made to improve the quality of research investigating the mental health impacts of COVID19 including recruiting more representative surveys, focusing more on understanding protective factors, and conducting longitudinal studies [6, 8]. A longitudinal study of US adults (using data collected from eight waves of the *Understanding America Study*, *n* = 7319) found that levels of distress (as assessed by the PHQ-4 [9]) increased in the early phase of the pandemic, before falling within a few months to pre-pandemic levels [10]. Similarly, a large nationally representative, longitudinal online study of UK adults (using data collected from six waves of the *UK Household Longitudinal Study*, *n* = 10,918) found that the prevalence of clinically significant psychological distress (as assessed by the GHQ-12 [11]) rose from pre-pandemic 2019 levels of 21% to 30% in April 2020, before then declining to pre-pandemic levels by September 2020 [12]. A further study investigated the trajectories of self-reported levels of anxiety (assessed by the GAD-7 [13]) and depression (assessed using the PHQ-9 [14]) experienced by adults across three time-points during a 20-week period (March 2020 to August 2020) of initial lockdown restrictions in England in a large (*n* = 36,520) prospective panel study [4]. Risk factors for comparatively high levels of depression and anxiety at the outset of the study included: being female, being a younger adult, having lower educational attainment, being in a low-income bracket, living alone or with children, and/or having a pre-existing mental health difficulty [4]. Levels of depression and anxiety decreased steadily across the duration of the study period, however those with pre-exiting mental health difficulties continued to have comparatively higher levels throughout [4]. The findings are suggestive of a process of psychological adaptation during the lock-down period [4]. More research is required to better understand what factors may be contributing to the purported process of psychological adaptation.

To date, there has been a comparative lack of research investigating the impact of COVID19 and associated restrictions on the Quality of Life (QoL) of general population samples. This is somewhat surprising because the assessment of QoL has been highlighted as an important indicator of global health (Epifanio et al., 2021) [15]. Of the limited studies that have been conducted, lower levels of QoL (as assessed by the WHOQoL) have been consistently shown to be associated with younger age, being female and having pre-existing physical health problems [15–17].

In a previous study we recruited a convenience sample of adults living in the UK (*n* = 600) via social media shortly after the UK government introduced a national lockdown early on in March 2020 [18]. A cross-sectional analysis of data collected in the initial wave found that having to self-isolate prior to the lockdown, feeling isolated, and having concerns about COVID19 impacting on one’s livelihood were linked to higher depression and anxiety scores (as assessed by the Hospital Anxiety and Depression Scale (HADS [19]), lower wellbeing (assessed using the World Health Organization (Five) Well-Being Index [20]) and lower quality of life (QoL) scores (as assessed by the OxCAP-MH [21]) [18]. The study highlighted the importance of employing multi-dimensional assessments of QoL (such as the OxCAP-MH) to assess a range of factors (including non-health issues, welfare inequalities etc.) that may be potentially important for understanding the impact of the pandemic and associated restrictions.

The development of the OxCAP-MH was influenced by the *Capability Approach* (CA) [22, 23]. The CA is a normative framework for social justice which focuses on individuals’ real freedoms (“*capabilities*”) to engage in forms of behaviour (“*functionings*”) that they have reason to value [22]. Whereas *capabilities* are what one is able to do or be (i.e. what is effectively possible), *functionings* are what one does or becomes (i.e. what is realised) [24, 25]. As such, the CA is concerned with understanding what individuals would wish to prioritize in terms of areas for personal development, their agency to pursue this agenda and the opportunities and resources that exist to realize this potential into actual, lived experiences. In the context of the global pandemic, a range of capabilities relating to health, education, housing, nutrition, and social connections have been substantially impacted [26]. A study conducted in Austria found that individuals who had tested positive or had symptoms of COVID19 reported decreased levels of capability-based QoL (assessed using the OxCAP-MH [21]) [27]. Overall, the authors concluded that the use of the CA is directly relevant to shedding light on the current public health emergency [27]. However, further longitudinal research is required to understand how capability-based QoL was impacted across the period of lockdown and how this was potentially associated with any changes in levels of common mental health difficulties.

The specific aims of the current study were to investigate whether capability-based QoL changed during the first 5-months (20 weeks) of lock-down restrictions in the UK, and whether any changes in capability-based QoL were predictive of changes in levels of depression and anxiety over time. We investigated the following hypotheses:

### Hypothesis 1

The levels of capability-based quality of life will increase, whilst levels of anxiety and depressive symptoms will decrease over the duration of the study.

### Hypothesis 2a

The levels of capability-based QoL will significantly predict additional variance in levels of anxiety when other variables (including age, gender, number of children and assessment time-point) are controlled for.

### Hypothesis 2b

The levels of capability-based QoL will significantly predict additional variance in levels of depression when other variables (including age, gender, number of children and assessment time-point) are controlled for.

### Hypothesis 3a

The levels of capability-based QoL at the study timepoints will predict future levels of anxiety symptoms, whereas the level of anxiety symptoms will not predict future levels of QoL.

### Hypothesis 3b

The level of capability-based QoL at the study timepoints will predict future levels of depressive symptoms, whereas the level of depressive symptoms will not predict future levels of QoL.

## Methods

### Study design

This study used a longitudinal design. Data from participants was collected at three timepoints over a 20-week period; the first data collection (T) took place between the 31^st^ of March and the 13^th^ of April 2020 – commencing one week after the introduction of the initial lock-down restrictions in the UK. The second round of data collection (T + 4) occurred approximately 4 weeks later between the 28^th^ of April and the 10^th^ of May 2020, when the lockdown was extended by the UK government. The third data collection (T + 18) took place approximately 18 weeks later between the 10^th^ and the 24^th^ of August 2020 when the lockdown restrictions were being eased. These timepoints were labelled to reflect the number of weeks since the first measurement was done. A timeline for the different lockdowns introduced in England (where the bulk of participants were living) is available here: https://www.instituteforgovernment.org.uk/sites/default/files/timeline-lockdown-web.pdf

### Participants

An opportunistic sample was used, participants were recruited through social media platforms (Twitter, Facebook and Reddit) during a two-week period (31^st^ March to 13^th^ April 2020) in the initial phase of COVID19 lock-down restrictions in the UK. To be eligible to participate, people were required to be a UK resident, understand written English, have access to the online survey.

At T, 594 participants completed the online survey, at T + 4, 222 participants completed the survey (37% attrition rate), and at T + 18, 147 people completed the survey (62% attrition rate). Independent samples t-tests were performed to test whether there were significant differences between those who participated in a wave of assessment and those that did not in terms of their scores on the HADS and/or OXCAP-MH at the preceding wave of assessment. These were all non-significant. Participants did not receive any reward or payment for participation. See Table 1 for sociodemographic data.

**Table 1:** Socio-demographic characteristics of participants

	***N at T *= 595 (%)**	***N at T+18* = 147 (%)**
Gender		
Female	443 (75%)	113 (77%)
Male	137 (23%)	27 (18%)
Non-Binary	9 (2%)	5 (3%)
Prefer not to say	2 (< 1%)	1 (1%)
Missing	3 (1%)	1 (1%)
Age (years)	M = 36.73	M = 41.23
	SD = 13.46	SD = 13.91
Ethnicity		
English/Welsh/Scottish/Northern Irish/British	520 (88%)	131 (89%)
Irish	11 (2%)	5 (3%)
Other White background	26 (4%)	7 (5%)
Chinese	2 (< 1%)	1 (1%)
Other	32 (6%)	3 (2%)
Missing	4 (1%)	
Country of residence		
England	532 (90%)	132 (90%)
Wales	19 (3%)	4 (3%)
Scotland	29 (5%)	6 (4%)
Northern Ireland	11 (2%)	4 (3%)
Missing	3 (1%)	1 (1%)
Education		
No qualifications	7 (1%)	4 (3%)
1–4 GCSEs or equivalent	16 (3%)	5 (3%)
5 + GCSEs or apprenticeship	25 (4%)	4 (3%)
A levels, vocational level 3 and equivalents	110 (19%)	24 (16%)
Higher education & professional/vocational equivalents	421 (71%)	107 (73%)
Other (including foreign)	8 (1%)	3 (2%)
No response	7 (1%)	0
Marital status		
Single, never married nor civil partnered	317 (53%)	72 (49%)
Married, including separated	205 (35%)	60 (41%)
Civil partnered, including separated	18 (3%)	3 (2%)
Divorced, including legally dissolved civil partners	39 (7%)	12 (8%)
Widowed, including surviving civil partners	3(1%)	0
Missing	2 (< 1%)	0
Children		
Yes	217 (37%)	62 (42%)
No	371 (62%)	85 (58%)
Prefer not to say	6 (1%)	0
Average number of children	M = 0.5	M = 2.23
	SD = 0.71	SD = 1.38
Employment status		
Student	108 (18%)	20 (14%)
Employed	390 (66%)	89 (61%)
Unemployed	46 (8%)	18 (12%)
Retired	33 (6%)	16 (11%)
Prefer not to say	17 (3%)	4 (3%)

### Materials and measures

At T, participants answered demographic questions and the following standardized assessment measures were used across all three timepoints:

#### Quality of life

The Oxford Capabilities Questionnaire – Mental Health (OxCAP-MH [21]) was used to assess capability-based QoL. It is a QoL questionnaire designed in the UK to capture different dimensions of QoL within the conceptual framework of the CA. The 16-item measure is scored on a 5-points Likert scale (Strongly agree, agree, neither agree nor disagree, disagree, strongly disagree), and items 2, 4, 5, 6, 9, 10, 11, 12, 13, 14, 15 and 16 are reverse coded. One of the items (item 8a) asks about the reasons for potential discrimination and is not included in the total score [20]. The OxCAP-MH is scored on a 16–80 scale, and the scores are converted (standardised) to a 0–100 scale, with higher scores indicating better capability-based QoL. Scores are converted using the formula: 100 × (OxCAP-MH total score – 16)/64 [20]. The OxCAP-MH had acceptable or good internal consistency at T (McDonald’s ω = 0.82), T + 4 (McDonald’s ω = 0.88), and T + 18 (McDonald’s ω = 0.87).

#### Anxiety and depression

The Hospital Anxiety and Depression Scale (HADS [19]) was used to measure anxiety and depression across timepoints. It is a 14-item measure of symptoms of common mental disorders that avoid reliance on somatic symptoms of depression and anxiety. All items are weighted equally on a 4-point Likert scale, where 0 reflects a positive extreme and 3 indicates a negative extreme. The HADS is divided into an Anxiety subscale (HADS-A) and a Depression subscale (HADS-D) both of which contain seven items, which are summed to obtain scores ranging from 0 to 21. For each subscale, scores are classified as normal (0–7 points), mild (8–10 points), moderate (11–14) or severe (11–21), therefore scores above 8 are considered high. The HADS had good/excellent internal consistency at T (McDonald’s ω = 0.90), T + 4 (McDonald’s ω = 0.94), and T + 18 (McDonald’s ω = 0.91).

### Procedure

When participants accessed the online survey on Qualtrics, the landing page was the participant information sheet (PIS). The PIS described what would happen during the study including a brief description of the survey. Participants were then asked to complete a consent form. If they did not give full consent online, participants did not gain access to the survey and instead were redirected to a ‘thank you’ page. If full consent was provided, they were directed towards the survey. At the end of the survey at T, participants were asked to provide an e-mail address to be contacted at a second timepoint (T + 4) and were then debriefed through Qualtrics. At T + 4, participants were also asked for consent to be contacted at T + 18 prior to being debriefed through Qualtrics. At all three timepoints, during the debrief participants were reminded of the aim of the study, the confidential nature of the research and the contact details of the researchers together with a list of national support organisations.

### Ethics

The authors assert that all procedures contributing to this work comply with the ethical standards of the relevant national and institutional committees on human experimentation and with the Helsinki Declaration of 1975, as revised in 2008. All procedures involving human participants were approved by the Central University Research Ethics Committees at the University of Liverpool (approval reference T: 7633, approval reference T + 4 and T + 18: 7688). Written informed consent was obtained from all participants at each of the three timepoints.

### Statistical analysis

#### Data estimation

Due to participant drop out (T_N_ = 594, T + 4_N_ = 222, T + 18_N_ = 147) missing data was estimated using multiple imputation (predictive mean matching) imputing 30 data sets (using all variables in the final regression models, anxiety, depression and, capability-based QoL, age, gender and number of children).

A linear mixed effects model for repeated measures data was conducted to estimate the changes for anxiety, depression and capability-based QoL over time. Furthermore, a linear mixed regression model was conducted with fixed slopes and participants as a random intercept. Capability-based QoL, sociodemographic factors (age, sex, number of children) and time (T, T + 4, and T + 18) were included as predictors in the model. The three timepoints were coded as a categorical variable. Continuous variables were mean centered. All analyses were performed using the lme4 (version 13.1093) package in R.

To estimate the directional influence of anxiety, depression, and capability-based QoL over time, a three-wave random intercept cross-lagged panel model (RICLPM) was created using Lavaan in R (version 13.1093). For the RICLPM the estimated data set. The RICLPM is a discrete-time structural equation modeling approach. In cross-lagged models, change in each variable over time is modelled using the autoregressive coefficients between time-adjacent measures of each variable (e.g. capability-based QoL at T predicts capability-based QoL at T + 4, and capability-based QoL at T + 4 predicts capability-based QoL at T + 18), and the cross-lagged effects between two variables (e.g. anxiety at T predicts capability-based QoL at T + 4, which in turn predicts capability-based QoL at T + 18). The random intercept allows for disaggregation of between person and within person effects, so by controlling for stable between subjects effects the autoregressive and cross lagged effects are indicative of within participant changes. In other words, a structural equation modelling approach was implemented in a cross-lagged model to evaluate the bidirectional relationships among anxiety, depression, and capability-based QoL over time.

The model fit was assessed by the ratio of the chi-square to degrees of freedom (*χ*^*2*^/*df*) using a Satorra-Bentler correction, the Tucker-Lewis Index (TLI), the comparative fit index (CFI), the standardized root-mean-square residual (SRMR), and the root mean square error of approximation (RMSEA). The CFI range between 0 and 1, with values higher than 0.90 indicating adequate model fit [28]. For the SRMR, a score of 0.08 indicated an acceptable fit, with a score approaching 0.05 indication superior fit [28]. RMSEA values below 0.06 were considered a good fit [28].

### Findings

#### Results for hypothesis 1

The mean scores for depression, anxiety, and capability-based QoL across the three timepoints and a linear mixed effects model for repeated measures data are shown in Table 2. The findings (see Table 2) indicate that both the levels of depression and anxiety significantly decreased across the three timepoints, indicating an improvement in mental health outcomes. On the other hand, there was no significant change in capability-based QoL over time.

**Table 2:** Linear mixed effects model for repeated measures of ANOVA for HADS anxiety, HADS depression and Capability-related QoL (*N* = 596)

	T	T + 4	T + 18	*F*	*p*	df	η_p_^2^	Bonferroni Post hoc test
HADS depression	7.59 (± 4.41)	6.86 (± 4.41)	6.45 (± 4.40)	12.54	< .001	2,1124	.02	T to T + 4 (*p* = .005) T to T + 18 (*p* < .002) T + 4 to T + 18 (*p* = .225)
HADS anxiety	10.23 (± 4.99)	9.77 (± 5.14)	9.37 (± 4.91)	5.75	.003	2,1124	.01	T to T + 4 (*p* = .0210) T to T + 18 (*p* = .002) T + 4 to T + 18 (*p* = .346)
Capability-related QoL	67.50 (± 12.92)	67.80 (± 13.13)	68.65 (± 12.68)	1.39	.249	2,1124	< .01	NA

#### Results for hypothesis 2a

##### ***Anxiety***

The mixed effect model was better than the single level model without a random intercept with ICC = 0.27 (χ^2^ (1) = 111.34, *p* < 0.001). As a first step time, age, gender, and number of children were added as predictors to the model. This was significantly better than the null model (χ^2^ (5) = 18.15, *p* < 0.001) and explained 32.23% of variance in anxiety at the participant level, and 1% at the measurement level. Subsequently, capability-based QoL was added as the next step, and this significantly improved the model (χ^2^ (1) = 209.41, *p* < 0.001), explaining an additional 20.69% of variance in anxiety at the single level, and 10.10% at the measurement level as compared to the first step. For each of the individual predictors see Table 3.

**Table 3:** Individual predictors of anxiety in a mixed effect model

Predictors	B	SE	95%CI	*p*
T + 4	-0.42	0.24	-1.47 – -0.28	0.085
T + 18	-0.71	0.24	-2.62 – -1.29	0.003
Age	-0.11	0.01	-0.14 – -0.05	< 0.001
Gender (m)	-1.06	0.30	-1.02 – 1.71	< 0.001
Children	0.45	0.13	0.11 – 1.04	0.001
OxCAP-MH	-0.11	0.02	-0.16 – -0.09	< 0.001

(Marginal R^2^ = .20)

#### Results for hypothesis 2b

##### ***Depression***

The mixed effect model was better than the single level model without a random intercept with ICC = 0.23 (χ^2^ (1) = 91.80, *p *< 0.001. As a first step time, age, gender, and number of children were added to the model as predictors. This was significantly better than the null model (χ^2^ (5) = 65.77, *p* < 0.001) and explained 12.23% of additional variance in depression at the participant level as compared to the null model, and 2.21% at the measurement level. Subsequently, capability-based QoL was included which significantly improved the model (χ^2^ (1) = 374.12,* p* < 0.001), and explained an additional 43.66% of variance in depression at the participant and an additional 15.43% of variance at the measurement level. For each of the individual predictors see Table 4.

**Table 4:** Individual predictors of depression in a mixed effect

Predictors	B	SE	95%CI	*p*
T + 4	-0.68	0.21	-1.09 – -0.27	0.001
T + 18	-0.96	0.21	-1.37 – -0.55	< 0.001
Age	-0.06	0.01	-0.08 – -0.04	< 0.001
Gender (m)	-0.23	0.25	-0.73 – 0.26	0.355
Children	0.39	0.11	0.18 – 0.61	< 0.001
OxCAP-MH	-0.15	0.01	-0.16 – -0.14	< 0.001

(Marginal R^2^ = .24)

### Random intercept cross lagged models

To examine the stability and relationship that capability-based QoL had with levels of anxiety and depression as assessed by the HADS over time (T, T + 4 and T + 18), two cross-lagged regression models were run.

#### Results for hypothesis 3a

##### Hads-A and capability-based quality of life

**Fig. 1:**
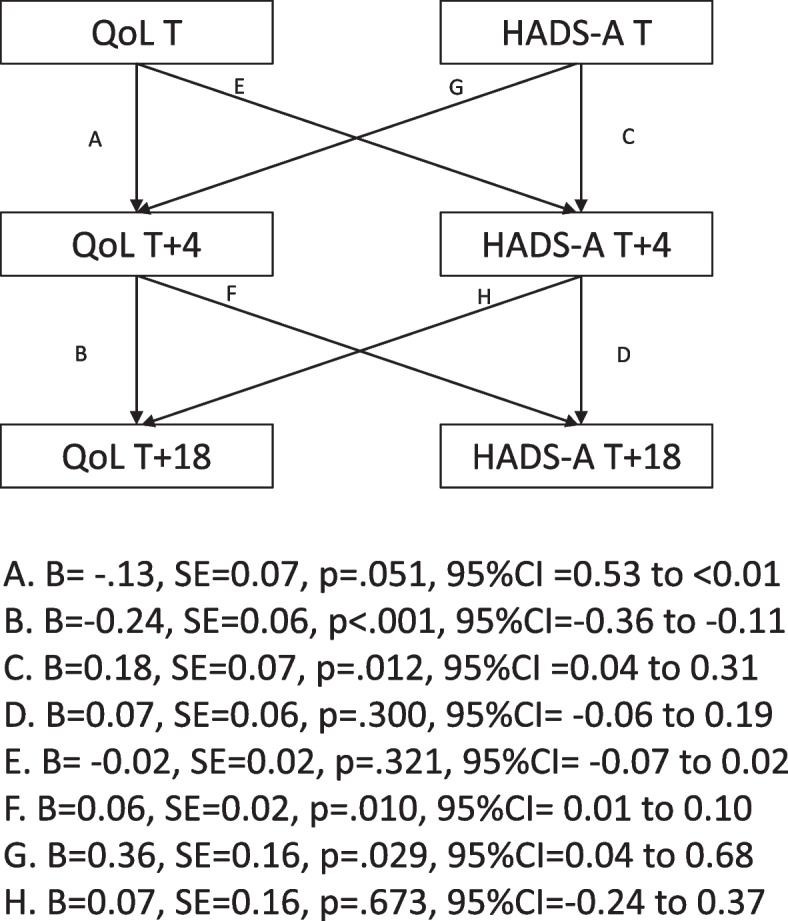
A cross-lagged regression model between anxiety and capability-based QoL

The initial model indicated high levels of covariance between anxiety and capability-based QoL at T + 4, therefore the model was rerun to fit the correlated residuals. The overall model fit was acceptable [CFI = 1.00, TLI = 1.00, SRMR = 0.007, χ^2^/df = 0.98) (see Fig. 1). There were significant cross-lagged relationships between levels of anxiety at T and capability-based QoL at T + 4, and between capability-based QoL at T + 4 and levels of anxiety at T + 18. No further significant cross-lagged relationships were found.

#### Results for hypothesis 3b

##### Hads-D and capability-based quality of life

**Fig. 2:**
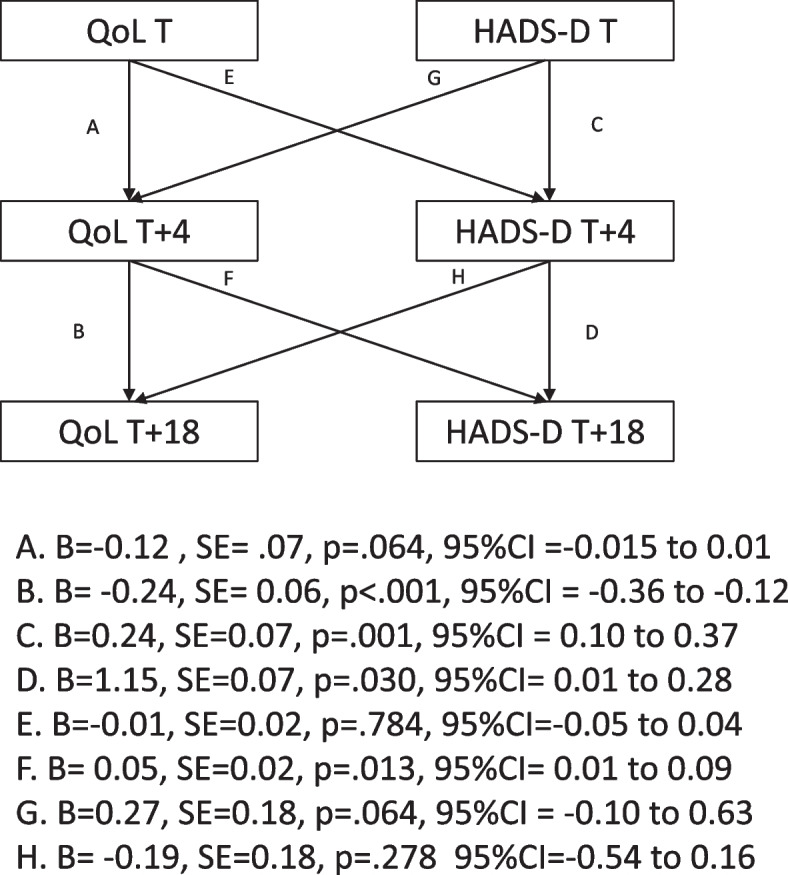
A cross-lagged regression model between depression and capability-based QoL

Similar to anxiety, the initial model indicated high levels of covariance between depression and capability-based QoL at T + 4, therefore the model was rerun to fit the correlated residuals. The cross-lagged model between depression and capability-based QoL showed a good model fit [CFI = 0.99, TLI = 0.99, SRMR = 0.008, χ^2^/df = 1.12).] (see Fig. 2). There was a significant cross-lagged relationship between capability-based QoL at T + 4 and levels of depression at T + 18. No further significant cross-lagged relationships were found.

## Discussion

The COVID-19 pandemic and associated lock-down restrictions have caused considerable disruption to people across the globe. There is a corresponding need to understand how these events have impacted on people’s mental health and wellbeing across time and what factors might be important for accentuating or mitigating risks. The current panel study collected data longitudinally in three-waves from a convenience sample of UK adults over a 5-month (20-week) time-period during the first COVID19 lock-down. The specific aims of the study were to investigate whether capability-based QoL changed during the first 5-months (20 weeks) of lock-down restrictions in the UK, and whether any changes in capability-based QoL (i.e. the extent to which people were free to do things that they have reason to value across multiple life domains) were predictive of changes in levels of depression and anxiety.

Hypothesis 1 was partially confirmed, as the findings indicated that, consistent with previous research focusing on depression and anxiety [4] specifically and distress more generally [9, 11], there was a pattern of improving mental health over the course of the 3 time-points. However, capability-based QoL did not significantly improve. Hypothesis 2a and 2b were confirmed, as the level of capability-based QoL was a significant predictor of both levels of depression and anxiety, and capability-based QoL predicted additional levels of variance in depression and anxiety when both time and sociodemographic factors were controlled for. This suggests that participants’ substantive freedom to do things that they have reason to value across multiple life domains (including performing usual activities, enjoying recreational activities, being free to express personal views etc.) was significantly associated with levels of anxiety and depression during the period of lock-down in the UK.

To ascertain causal links between capability-based QoL and levels of depression and anxiety (i.e. hypotheses 3a and 3b) random intercept cross-lagged panel model (RICLPM) analyses were conducted. These RICLPM partially confirmed hypothesis 3a, which looked at whether the levels of capability-based QoL at the study timepoints predicted future levels of anxiety symptoms (and not vice-versa). Contrary to what we predicted, levels of capability-based QoL at the outset of the lockdown restrictions (i.e. T) did not predict levels of anxiety just over a month later (T + 4). Instead, the levels of anxiety at T predicted levels of capability QoL at T + 4. However, participants’ capability-based QoL at T + 4 did predict levels of anxiety at T + 18. Similarly, hypothesis 3b was also partially supported. Contrary to what was hypothesized, the level of capability-based QoL at baseline did not predict levels of depression scores just over a month later (T + 4). However, capability-based QoL at T + 4 did predict depression scores at the subsequent wave of assessment (T + 18).

These findings suggest that just over a month (i.e. T + 4) following the introduction of lockdown restrictions, participants’ perception of having greater substantive freedoms to do things that they have reason to value across multiple life domains predicted subsequent levels of both anxiety and depression approximately 5 months into the lockdown (T + 18). The direction of the relations is important to note. It appears that participants with higher capability-based QoL a month into the lockdown experienced higher levels of depression and anxiety as the lockdown progressed. Although we can only speculate about the reasons for this pattern, it may be that individuals who had higher levels of capability-based QoL just over a month into the first lock-down experienced a more profound emotional impact as the restrictions persisted and their ability to maintain capability-based QoL was curtailed. It is unclear why high levels of anxiety one week into the lockdown restrictions might have predicted higher levels of capability-based QoL four weeks later. It is possible that high levels of anxiety early in the lockdown had the effect of mobilizing people to do their utmost to maintain capability-based QoL across different life domains in spite of the restrictions. However, these claims are speculative and other factors may have contributed to this.

The findings provide support for the notion that public health emergencies such as COVID19 can impact on people in different ways. Rather than the lockdown restrictions per se being important for mental health and wellbeing, it may be the extent to which people perceive the restrictions to have a capability-limiting impact on their lives that that is associated with their levels of depression and anxiety. The findings of the current study have several important implications for both policy and service provision in relation to providing support in the context of public health emergencies that may restrict people’s freedoms. The distinction between *form* vs. *purpose* of behaviour seems important here. Whilst behaviours can serve a particular *purpose* (e.g. maintaining one’s physical fitness)*,* they can assume a variety of different *forms* (going to a gym, taking a walk outside, cycling on an exercise bike at home). From a CA perspective, the imposing of restrictions may lead to reduced opportunities to engage in particular forms of behaviour, however opportunities may well still exist to develop a particular capability through engaging in alternative behaviours that serve the same purpose. Indeed, it has been suggested that government responses that focused on safeguarding and maintaining capabilities, rather than alternative metrics such as income, were more successful [29]. Future research should investigate what types of psychological support can help expand different peoples’ capability sets, increase their capability-based QoL, and reduce their levels of anxiety and depression with a particular focus on how different contexts affect peoples’ responses during public health emergencies.

Although the current study was able to commence at a very early stage of the pandemic lockdown in the UK and prospectively followed up a sample of adults, there are several methodological limitations that need to be acknowledged. Firstly, we recruited a convenience sample via social media channels. The absence of a sampling framework and the reliance on participants being technologically proficient to access the online mode of participation means that the sample recruited is not representative of the population at large. As such, the findings may not generalise well to the UK public. Secondly, given the rapidly changing situation during the global pandemic, we did not conduct an a priori power analysis. Ideally, we would have undertaken a pilot study to generate effect sizes and then conduct a simulation on this data. But this was not possible because the research was time sensitive. Instead, we aimed to maximise recruitment within time constraints. As a result, the sample size was comparatively small. However, it is important to note that the number of predictor variables included in the analyses were not excessively high. There was attrition at each of the three waves of assessment. It is possible that those who were most profoundly impacted during the lock-down, or indeed those who were least impacted, may have had cause not to participate in subsequent rounds of assessment. Independent samples t-tests, however, did not indicate that there were significant differences between those who participated in a wave of assessment and those that did not in terms of their scores on the HADS and/or OXCAP-MH at the preceding wave of assessment. Thirdly, limitations in relation to RICLPM analyses of the type used in the current study have been highlighted. For example, it has been suggested that “if observations within a study are gathered with unequal time-intervals, the standard CLPM will lead to biased results, because it will model the unequal time-intervals as if they were equidistant. (a.o [30].)” [31]. The duration between the start and end assessment points for the three waves of data collection in the current study were not equidistant (T to T + 4: 4–6 weeks; T + 4 to T + 18: 14–16 weeks), as this was determined by the restrictions imposed by the national government. Therefore, although the repeated assessment is a strength of the current study as it allowed for an evaluation of changes within each construct and how these changes influenced each other, some research would argue for the use of continuous time modelling. The ‘Continuous-Time’ modelling approach has been proposed as a way of addressing the difficulties posed by the time-interval dependency issue [32, 33] and should be used in future research with larger samples.

## Conclusion

Consistent with other longitudinal studies, the current study provided a picture of improving levels of depression and anxiety symptoms during the initial 5-months (20 weeks) of the UK lockdown. Uniquely, the current study found that over a month into the lock-down restrictions people’s level of capability-based quality of life seems to be an important contributing factor to the levels of depressive and anxiety symptomatology that they experienced 5 months into the lockdown restrictions. Concerted efforts should be made to help people to think flexibly and creatively about how they can utilize a range of approaches to maintain capability-based quality of life.

## Data Availability

The data that support the findings of this study are available from the corresponding author upon reasonable request.
